# Improving automatic GO annotation with semantic similarity

**DOI:** 10.1186/s12859-022-04958-7

**Published:** 2022-12-12

**Authors:** Bishnu Sarker, Navya Khare, Marie-Dominique Devignes, Sabeur Aridhi

**Affiliations:** 1 CNRS, Inria, LORIA, University of Lorraine, 54000 Nancy, France; 2 Khulna University of Engineering and Technology, Khulna, Bangladesh; 3 International Institute of Information Technology, Hyderabad, India; 4 School of Applied Computational Sciences, Meharry Medical College, Nashville, TN USA

**Keywords:** Protein function annotation, Domain similarity network, Gene ontology annotation, Label propagation, Semantic similarity, GrAPFI, K-nearest neighbor

## Abstract

**Background:**

Automatic functional annotation of proteins is an open research problem in bioinformatics. The growing number of protein entries in public databases, for example in UniProtKB, poses challenges in manual functional annotation. Manual annotation requires expert human curators to search and read related research articles, interpret the results, and assign the annotations to the proteins. Thus, it is a time-consuming and expensive process. Therefore, designing computational tools to perform automatic annotation leveraging the high quality manual annotations that already exist in UniProtKB/SwissProt is an important research problem

**Results:**

In this paper, we extend and adapt the GrAPFI (graph-based automatic protein function inference) (Sarker et al. in BMC Bioinform 21, 2020; Sarker et al., in: Proceedings of 7th international conference on complex networks and their applications, Cambridge, 2018) method for automatic annotation of proteins with gene ontology (GO) terms renaming it as GrAPFI-GO. The original GrAPFI method uses label propagation in a similarity graph where proteins are linked through the domains, families, and superfamilies that they share. Here, we also explore various types of similarity measures based on common neighbors in the graph. Moreover, GO terms are arranged in a hierarchical manner according to semantic parent–child relations. Therefore, we propose an efficient pruning and post-processing technique that integrates both semantic similarity and hierarchical relations between the GO terms. We produce experimental results comparing the GrAPFI-GO method with and without considering common neighbors similarity. We also test the performance of GrAPFI-GO and other annotation tools for GO annotation on a benchmark of proteins with and without the proposed pruning and post-processing procedure.

**Conclusion:**

Our results show that the proposed semantic hierarchical post-processing potentially improves the performance of GrAPFI-GO and of other annotation tools as well. Thus, GrAPFI-GO exposes an original efficient and reusable procedure, to exploit the semantic relations among the GO terms in order to improve the automatic annotation of protein functions

## Background

More and more protein sequences are accumulating in public databases thanks to advanced high-throughput sequencing technology [3]. To get valuable information from this vast amount of data, we need to associate appropriate functional properties for these protein sequences. Automatic prediction of functions of uncharacterized proteins is an important topic in the field of bioinformatics, as manual function identification methods are costly and time consuming. This poses many challenges for both biologists and computer scientists. Complete knowledge of the functional properties of proteins is central to understanding life at the molecular level and is the key to understanding human disease processes and drug discovery efforts. [4]. The UniProt knowledgebase (UniProtKB), the most comprehensive publicly available protein database, consists of two parts: i) the UniProtKB/Swiss-Prot database contains the manually reviewed protein sequences [3, 5] and ii) the UniProtKB/TrEMBL database is used for storing un-reviewed protein sequences [5]. According to the $2021\_01$ release of UniProtKB, some 564,000 sequences have received manually reviewed functional annotations, whereas nearly 208 million protein sequences lack reviewed functional annotations. In UniProtKB, protein information includes primary sequence as well as some other attributes such as the structural domains and family information. These attributes can be explored to compute pairwise similarity among proteins. However, concerning function annotation, proteins in UniProtKB/TrEMBL only display limited results from automatic tools and lack reviewed information in contrast to UniProtKB/Swiss-Prot proteins that are always annotated with well reviewed functional attributes. The huge quantity of TrEMBL proteins calls for efficient and rapid procedures to annotate them automatically.

Gene ontology (GO) [6] lists controlled vocabulary of terms that denotes the functional attributes and these terms refer to the various functions that the proteins and genes are performing in our body. In GO, functional terms are presented in a hierarchy in three different directed acyclic graphs (DAG) namely (1) Biological Process (BP), (2) Molecular Function (MF), and (3) Cellular Component (CC). Each GO term is a node in the DAG. Every DAG starts from a root term and it connects to other terms hierarchically through various types of links indicating different types of relationships. The most commonly used relationships are “is a”, “part of”, and “regulates” as appears in the GO database.

Annotation by association rules based on sequence and structural similarities is still the dominating approach for automatic protein function annotation problem. The central idea is to find the statistically significant pair-wise similarities between un-annotated query protein and the annotated proteins stored in the database and then transferring the known annotations from the highly ranked similar proteins. That is, when a new protein arrives in the database, it is matched against known proteins in the database and the known annotations are transferred from the best matched proteins.

UniProtKB managed by European Bioinformatics Institute (EBI) deploys automatic annotations tools for accelerating the annotation of incoming un-annotated proteins. Primarily, a rule based system called UniRule [7] is used by the UniProtKB to annotates the proteins using manually curated, expertly defined rules of the form “if-then”. Traditionally, expert biologist and curators gather around experimental evidences reading published scientific articles and literature, and complementing their domain expertise, they decide on Criteria to be used to annotate a protein. Once few of the proteins are manually annotated using following these criteria, the become rules and they serve as the seeds to devise the UniRule rules. As it is evident from the description, these rules are manually validated by experienced curators. UniRule rules can annotate protein properties such as the protein name, function, catalytic activity, pathway membership, and sub-cellular location, along with sequence specific information, such as the positions of post-translational modifications and active sites. In general, UniRule rules are very reliable. However, this is a laborious and time consuming process that very much depends on human expertise. Moreover, and although the exact number is not readily available from UniProtKB, it is likely that the UniRule annotation coverage is very low compared to the rate of accumulation of proteins in UniProtKB/TrEMBL.

To alleviate difficulties with curating manual rules in UniRule, data-driven machine learning techniques have been put into the annotation pipeline. The system is called Statistical Automatic Annotation System (SAAS) [8] and it acts as a complementary system to support the labour-intensive UniRule system by generating automatic annotation rules. SAAS generates automatic annotation rules using the decision tree algorithm [9] applied over the already annotated entries in UniProtKB/SwissProt.

In August 2020, SAAS was replaced by another updated rule-based system called ARBA (Association Rule-Based Annotator) [10–12], which is now the mainstream annotation pipeline in UniProtKB to annotate UniProtKB/TrEMBL protein entries. ARBA is a multi-class machine learning system trained with expertly annotated entries in UniProtKB/Swiss-Prot. It uses association rule mining to identify potential rules that are associated with certain types of function. It generates precise annotation models i.e. rules and performs with relatively higher accuracy and coverage.

Along with functions, ARBA rules can annotate other protein properties such as catalytic activity, pathway membership, sub-cellular location and protein names. It generates around 23 thousands models/rules that associates InterPro signatures and taxonomic identities to corresponding functions and the rules are learned completely from the annotated entries of UniProtKB/SwissProt.

Various attributes were also used for protein function annotation. In [13–16], structural similarity of proteins is used for the purpose of function prediction. In [17–22], homology relationships s used to annotate un-reviewed sequences. In addition, several machine learning based techniques are extensively studied in [14, 23–36].

Network science [37] is perceived as the science of analyzing linked data, the data that is presented as a network consists of nodes and connections. Due to its prolific importance in explaining complex real-world inter-connected systems, network science has become a successful inter-disciplinary area of research. Network science is an applied domain finding applications in diverse real-world use cases ranging from banking and the internet routing to modeling the human brain and understanding complex biomedical process. In the literature such as [2, 38–42], we find many such researches that demonstrate the usability of network science in the context of automatic protein function annotation. Most prominent among the approaches is neighborhood based techniques for protein-to-protein propagation of functional information using protein–protein interaction (PPI) networks and Gene Ontology terms. The objective is to explain the functional similarity depending on the interactions in the PPI network. Biological networks are complex partly because they encode complex biological entities like proteins. Therefore, decoding biological networks often requires expert biological knowledge to fully understand and exploit the network.

In pursuit of developing automatic protein function annotation tools, a number of pipelines are proposed.These tools exploit various attributes for example, sequence encoding, functional domain similarity, structural similarity, phylogenetic tree, protein–protein interaction network etc. Followings are brief description of a few state-of-the-art GO prediction tools.

*Blast2GO or B2G* [43] is a close source software suit that harnesses the sequence similarity for the purpose of functional annotation using GO terms. BLAST [44] is a popular and fast sequence searching tool that is being used at the first step to retrieve matched sequences along with associated GO terms together with their evidence code which is interpreted as an index of the trustworthiness of the GO annotation. To find specific annotations with certain level of reliability, an annotation score (AS) is computed for each candidate GO term, which is composed of two additive terms. The first direct term (DT), represents the highest hit similarity of this GO term weighted by a factor corresponding to its evidence code. The second term (AT) of the AS provides the possibility of abstraction. This term multiplies the number of total terms unified at the node by a user-defined GO weight factor that controls the possibility and strength of abstraction. Finally, lowest terms per branch that lie over a user-defined threshold are selected. Once GO annotation is available through B2G, the application offers the possibility of direct statistical analysis on gene function information. Although this tool is serving the goal fairly effectively based on the standard evaluation metrics, it is not open source and free to use that effectively limits its use to the wider community.

*GoFDR* [45] is a sequence alignment-based algorithm that align a sequence with a group of sequence for the purpose of relating them evolutionarily. It takes a query sequence as input and then runs BLAST [44] or PSI-BLAST [46] to compute multiple sequence alignment (MSA) against a large set of protein sequences coming from a protein database. In the next step, it gathers around associated GO terms of the proteins participated in the MSA process. Functionally discriminating residues (FDRs) are computed for each GO term which then used to build a position-specific scoring matrix (PSSM). PSSM is used to compute the score between the query protein and a GO term, followed by a raw score adjustment step to convert the raw score into a probability. Multiple sequence alignment is an computationally expensive step in the proposed techniques difficult to run in a low resource set-up.

*DeepGO* [47] is a deep-learning-based technique. It leverages the power of deep learning to compute low-dimensional feature representation using protein sequences as well as cross-species protein–protein interaction (PPI) network. It utilizes the dependencies between GO classes as background information to construct a deep learning model. The input of the model is amino acid (AA) sequence of proteins. The sequence is transformed into a list of 3-mers - setting a cursor pointing to the first AA, 3 AAs taken consecutively while the cursor is moved by 1. As there are 20 amino acid, there is 8000 3-mers possible mathematically. Using a dictionary of size 8000, it is then represented as one-shot encoding vectors and feed into the deep learning model followed by a dense embedding layer. A 1D convolution is applied over protein sequence data and redundant information from the resulting feature map is discarded through temporal max-pooling. Additionally, applying knowledge graph embedding on multi-species PPI networks, DeepGO finds another feature representation for the proteins which are then combined with the output of the max-pooling layer to form a combined feature vector. Finally, fully connected layers for each class in GO are used to create a hierarchical classification neural network model that encodes transitivity of subclass relations. The main advantage of this approach is that it does not rely on manually crafted features and is therefore an entirely data-driven approach.

*PANNZER* [48, 49] applies a machine learning technique, specifically weighted k-nearest neighbours to predict the functional annotation of proteins. It performs a sequence similarity search against sequence database to obtain a Sequence Similarity Result List (SSRL). To reduce searching biases, much of the attention is put only on the sequences having strongest results from sequence scoring by setting filtering thresholds on alignment coverage, identity percentage, sequence length and informative descriptions. Non-linear weighting of taxonomic distances is another source of information used in PANNZER, corrected with a non-linear similarity function between the descriptions of compared query and target sequence. In the second step, PANNZER pipeline applies sparse regression model to re-score the sequence hits by combining various signals from sequence alignment and non-linear taxonomic distance score. The weighted sum of score functions computed is optimized against weighted similarity. In the final regression model all terms that had negative correlation with predicted variable from the model are excluded and final score is obtained.

*COFACTOR* as described in [15, 16] is mixed approach that combines sequence and structural information together for protein function annotation. In another more recent version, **COFACTOR** [50] integrates Protein–Protein Interaction (PPI) networks with information about protein structure and sequence homologs to build a hybrid model for jointly predicting different functional characteristic such as GO terms, EC numbers, and ligand-binding. The query protein sequence that is provided as an input is translated into a 3-D structure with the help of in-house 3-D structure prediction tool. In the next step, a template matching algorithm is applied to search for the nearest homologs with the highest structural similarities. By following similar principle, the sequence homologs are also searched by using BLAST software. And finally the interacting proteins are discovered from PPI network. These three elements constitutes the foundation for transferring the annotation from homologous proteins to the query protein.

In this paper, we extend the GrAPFI method [1, 2], a Graph-based Automatic Protein Function Inference approach, to the GO annotation, renaming it **GrAPFI-GO**. We propose a pruning technique based on semantic similarity to eliminate the outlier annotations and a hierarchical post-processing step to enrich the remaining annotations with term ancestors. More specifically, our contributions are the followings:

- We extend GrAPFI to perform GO Prediction. GrAPFI is a neighborhood-based label propagation approach that works on a network of proteins connected using domains and family information. GrAPFI was originally proposed for Enzymatic protein function prediction using Enzyme Commission (EC) Number.
- We incorporate various approaches for computing node similarity based on common neighbors to take into account the local graph sub-structures around each node of a pair.
- We integrate semantic similarity to take into account the hierarchical nature of the GO data and prune outlier annotations based on their distance in the GO semantic space. To find functional similarity, we used GOGO [51] which is claimed to be a fast and efficient way of computing GO term similarity. We also enrich the pruned list of GO terms with term ancestors.
- We experimentally evaluate the performance of the proposed approach by annotating a benchmark of protein sequences with GO terms and report a comparative analysis of the efficacy of the proposed pruning and post-processing technique for GO term prediction.

## Results and discussion

### Data preparation

In order to validate the performance of the proposed method, we have used a dataset of 1000 proteins [52]. For GrAPFI, we build the network using the training data from CAFA3, the 3rd edition of CAFA.^1^ CAFA3 is a well known competition that aims to annotate protein sequences. Usually, CAFA3 provides target and training sequences to build annotation models. In this study, to build the network, we have used CAFA3 training sequences and we have collected domain and family information for those proteins from UniprotKB. Then, we have built the graph of CAFA3 training proteins. This graph contains around 65,000 nodes as proteins and an average of 16 ground-truth GO terms per protein.

To prepare the test set, we have used MetaGO benchmark sequences and run InterProScan [53] to identify the domains and family information from the sequence. Using the domains and family information of test proteins, we run GrAPFI-GO and other annotation tools over all test proteins and retrieve predicted annotations. We then apply or not the proposed semantic pruning and hierarchical post-processing method and we compare the efficiency of annotation using standard metrics.

### GO annotation performance analysis

In Table 1, we present average precision, recall and F1-score for GrAPFI-GO on all proteins of the MetaGO benchmark in 9 different scenarios. Firstly, GrAPFI-GO is run on MetaGO benchmark for GO annotation and common neighbor similarity is not used during label propagation. For comparison, the values reported for F1-score in the MetaGO study were 0.391, 0.454, and 0.589 for BP, MF, and CC respectively, at the 20% cutoff in sequence identity. Thus the GrAPFI-GO method displays about half the MetaGO performance, which can be explained by the absence of requirement for structural modelling in GrAPFI-GO. This relatively low performance of GrAPFI prompted us to develop strategies for improving the method, as described in this study.

In the rest of Table 1, GrAPFI-GO is run on MetaGO benchmark for GO annotation and eight different neighbor similarity measures are included during label propagation : number of common neighbors (Eq. 2; GrAPFI-CN), Jaccard index (Eq. 3; GrAPFI-JA), preferential attachment index (Eq. 4; GrAPFI-PA), Salton index (Eq. 5 : GrAPFI-SA), Sorensen index (Eq. 6; GrAPFI-SO), hub depressed index (Eq. 8; GrAPFI-HDI), hub promoted index (Eq. 7; GrAPFI-HPI), local Leicht–Holme–Newman index (Eq. 9 ; GrAPFI-LLHN). In each case, precision, recall and F1-score are calculated separately for each GO aspect : (1) biological process, (2) molecular function and (3) cellular component, and averaged over all the proteins of the benchmark. The results show that GrAPFI-LLHN performs slightly better than other neighbor similarity methods. However, the equally better scores of GrAPFI-GO without common neighbor similarity discourage the added complexity in the annotation process. The results indicate that taking into account the first-order neighbors in the label propagation does not significantly improve the outcome. Additionally, it incurs a high computational cost.

**Table 1:** Comparison of GrAPFI-GO performance with and without node neighborhood-based similarity

Method	GO aspects	Precision	Recall	F1-score
GrAPFI	BP	0.28	0.21	0.24
	MF	0.23	0.28	0.25
	CC	0.33	0.31	0.32
GrAPFI-CN	BP	0.26	0.20	0.23
	MF	0.26	0.21	0.23
	CC	0.32	0.30	0.31
GrAPFI-JA	BP	0.26	0.19	0.23
	MF	0.21	0.27	0.24
	CC	0.32	0.30	0.31
GrAPFI-PA	BP	0.25	0.19	0.22
	MF	0.21	0.26	0.23
	CC	0.31	0.29	0.30
GrAPFI-SA	BP	0.26	0.20	0.23
	MF	0.21	0.26	0.23
	CC	0.33	0.30	0.31
GrAPFI-SO	BP	0.26	0.20	0.23
	MF	0.21	0.26	0.23
	CC	0.33	0.30	0.31
GrAPFI-HDI	BP	0.26	0.19	0.22
	MF	0.21	0.26	0.23
	CC	0.32	0.30	0.31
GrAPFI-HPI	BP	0.26	0.19	0.22
	MF	0.21	0.26	0.23
	CC	0.33	0.30	0.31
GrAPFI-LLHN	BP	0.28	0.21	0.24
	MF	0.22	0.27	0.24
	CC	0.33	0.31	0.32

GrAPFI-CN: common neighbor, GrAPFI-JA: Jaccard index, GrAPFI-PA: preferential attachment, GrAPFI-SA: Salton index (), GrAPFI-SO: Sorensen index, GrAPFI-HDI: hub depressed index, GrAPFI-HPI: hub promoted index, GrAPFI-LLHN: local Leicht–Holme–Newman index. In each case, we report average precision, recall and F1-score for three GO aspects, namely Biological Process (BP), Molecular Function (MF) and Cellular Component (CC). The experiment is run with cut-off score of 0.5, minimum similarity threshold of 0.3

In Table 2, we evaluate the effect of adding the semantic pruning and hierarchical post-processing steps on the annotation performance of GrAPFI-GO and two other recently developed annotation tools.

Among the top performing methods, only a few have their source code available to run experiments. Therefore, we focus on two easily available tools namely PANNZER and DeepGOPlus [54], an improved version of DeepGO. DeepGoPlus learns models with less parameters than DeepGO. These tools have been recently published and are claimed to be high performing.

We run the three selected annotation tools on the MetaGO benchmark data and obtain sets of predicted annotation terms. These predicted sets are further pruned using semantic similarity and enriched with hierarchical post-processing. Results are shown in Table 2. Here, we present evaluation metrics without categorizing into GO aspects as the multi-step pruning and post-processing method takes huge time to produce the predicted annotation set. For each test protein, Model Score (MS, provided by each method) and semantic similarity score (SS, calculated with our implementation of GOGO) are obtained for all predicted terms for this protein. Different cut-offs of these two scores are used for analysis. The final set of predicted terms is compared to the ground-truth in the benchmark. For each annotation tool, Table 2 shows the annotation performance in four cases: (1) without any kind of pruning and post processing, (2) when the highest SS score is the cutoff for SS, (3) when the 5th highest SS score is the cutoff for SS and (4) when the 5th highest SS score and half of the maximum MS score are the cutoffs for SS and MS respectively.

From Table 2, it appears clearly that the proposed post-processing approach that uses the semantic similarity of predicted GO terms to prune the predicted set as well as ancestor enrichment, significantly improves the overall performance of the GrAPFI-GO method. In particular, it improves the precision by many folds, jumping from 16.5% to 57.3% when using maximum SS as cut-off during post-processing. Similarly, the proposed approach improves the precision of PANNZER and DeepGOPlus by many folds. However, for these two methods, it leads to reduced recall values as the number of predictions gets much lower than the ground-truth predictions. This reduced number of predictions per protein essentially reduces the recall score and this ends up having lower F1-score for the PANNZER tool. Interestingly, the F1-score is by contrast enhanced for the DeepGOPlus tool despite of a decreased recall but thanks to the 3- to 5-fold increase in the precision value.

**Table 2:** Effect of pruning and post-processing method on the performance of GrAPFI-GO and two other automatic annotation tools

Method	Post-processing cut-off	Precision	Recall	F1-score
GrAPFI	No-post-processing	0.165	0.108	0.107
	SS-max	**0.573**	0.115	0.175
	SS-5	0.445	0.380	0.376
	SS-5-MS-max/2	0.440	**0.391**	**0.379**
PANNZER	No-post-processing	0.547	**0.942**	0.668
	SS-max	**0.637**	0.225	0.301
	SS-5	0.634	0.515	0.536
	SS-5-MS-max/2	0.603	**0.689**	**0.609**
DeepGOPlus	No-post-processing	0.053	**0.653**	0.095
	SS-max	**0.249**	0.120	0.138
	SS-5	0.186	0.182	0.160
	SS-5-MS-max/2	0.167	0.233	**0.1725**

The bold numbers indicate which post-processing cut off achieved the maximum performance score for a particular performance metric and annotation tool

Average precision, recall and F1-score are computed for each method in four situations. No-post-processing: without post-processing and pruning; SS-max: pruned using highest SS as cut-off; SS-5: pruned using 5th highest SS as cut-off; SS-5-MS-max/2: pruned using 5th highest SS and (maximum MS)/2 as cut-offs

## Conclusion

Automatic protein function annotation is an important topic in the field of bioinformatics because of the lack of annotation of proteins due to high costs and time-consuming nature of manual procedures for function identification. There exist a number of tools to perform automatic protein function annotation using GO terms, EC numbers, ligand binding sites etc. These tools use various attributes and different methods to accomplish the task. Although they show higher performance based on F1 score, the high F1 score is coming from a higher recall as they predict a large number of candidate annotations. This, in turn, increases the number of false positive annotations. In this paper, (1) we present GrAPFI-GO, a graph-based protein function inference method for GO term prediction, (2) We incorporate node similarity scores with link weight to use sub-structures among the neighbors, and (3) we propose an efficient pruning and hierarchical post-processing technique by integrating semantic similarity of candidate annotations. We experimentally validate that the proposed method can significantly improve the annotation outcome. In fact, for the two tools tested in addition to GrAPFI-GO, recall is significantly lower as the number of annotations decreases compared to the initial number of annotations predicted by these tools. Nevertheless, the precision is improved by many folds as the post-processing method helps to select highly coherent semantically close annotations. Any of the available annotation tools can benefit from the proposed post-processing approach. Therefore, any improvement in the automatic annotation pipeline would be magnified with the proposed hierarchical post-processing.

Functional annotation of protein is a challenging problem equally in the case of manual and automatic pipeline. In the manual annotation process, curating rules is a difficult step. In case of automatic annotation pipeline, the models are trained with insufficient ground truth knowledge. And, as the number of manually reviewed proteins increases in every release, more facts are being available to be used in the automatic prediction methods. This eventually provides enhanced training data. On the other hand, due to fast progress in computing systems, this is now possible to perform large scale complex annotation pipeline.

The work presented in the paper, rather, presents promises of the proposed approaches. A practically relatable large scale experiment would require powerful computing systems, distributed processing, and large biologically significant training data along with advanced AI techniques merged with expert rules. It would be also interesting to explore other semantic similarity approaches to establish the efficacy of the pruning and hierarchical post-processing techniques. There is also scope of using advanced representation learning for computing low rank vector representations or embeddings of the GO terms. These embeddings eventually can be utilized to compute semantic similarity and thus, develop new membership scores for the GO terms.

The automatic protein function annotation is a big support to the protein annotation process. However, this is not the complete alternative of the manual process.

## Methods

GrAPFI-GO—the proposed the automatic protein annotation technique—performs GO annotation using a neighborhood-based label propagation proposed in GrAPFI combined with hierarchical post-processing to take advantage of the hierarchical organization of Gene Ontology. Essentially, there are two parts in the pipeline: (1) predicting the set of GO terms using GrAPFI, and (2) pruning the annotation set using semantic similarity and hierarchical post-processing. In its original form, GrAPFI build graph of proteins exploiting their similarity in domain composition and applies neighborhood-based label propagation to automatically annotate proteins with EC number. In the current form, GrAPFI is extended as GrAPFI-GO for protein function annotation using Gene Ontology (GO) terms.

More specifically, the objective is to develop a pipeline that will leverage the functional annotations of manually reviewed proteins in Swiss-Prot to predict those of non-reviewed or un-annotated proteins in TrEMBL by building a graph of proteins and then applying function inference technique on the graph. The GrAPFI algorithm first constructs an undirected weighted graph of the proteins using the domain composition of the annotated proteins. Each protein is a node and a link exists if there is similarity in the domain composition between two proteins.

Then, given an non-annotated protein, a label propagation algorithm is applied to the domain similarity graph in order to infer appropriate annotations.

### Notation

In this section, we first present some definitions and notations used in the paper.

*Graph* Graph is a data structure to represent relations and connections among objects and entities. Symbolically, a graph is defined as a collection of vertices and edges denoted by $G = (V,E)$, where *V* contains the set of vertices/nodes and $E \subseteq V \times V$ is the set of edges.

*Weighted graph* The links or edges in a graph may have weights for quantifying the bonding. Such graph is called weighted graph. In a weighted graph, in addition to *V* and *E*, there is another component *W* to hold the weights of the edges. Symbolically,the graph is denoted as $G= (V,E,W)$ where:

- *V* is a set of nodes,
- $E \subseteq V \times V$ is a set of edges,
- *W* is a weight matrix where each cell $W_{uv}$ represents a numerical weight of the edge $(u,v)\subseteq E$.

*Labeled graph* In some settings, graphs contain labels for vertices or edges exposing the class of the nodes and type of relations that the edges are holding. THis type of graphs are termed as labelled graph. The notation to represent a labeled graph $G= (V,E,L,I)$ where:

- *V* is a set of nodes,
- $E \subseteq V \times V$ is a set of edges or links,
- *L* is a set of labels,
- $I : V \cup E \longrightarrow L$ is a mapping function that put appropriate labels from *L* to nodes *V* and edges *E*.

*Directed graph* In a directed graph, the direction of the edges are explicitly expressed by specifying the source and destination vertices of the edges. $G = (V,E)$ is a directed graph if $E \subseteq V \times V$ contains set of edges with ordered pair of vertices (*u*, *v*) such that $(u\longrightarrow v) \in E$.

*Undirected graph* In an undirected graph, the direction of the edges is not explicitly mentioned. $G = (V,E)$ is undirected graph if $E \subseteq V \times V$ is a set of edges with unordered vertices (*u*, *v*) such that if $(u\longrightarrow v) \in E$ exists then $(v\longrightarrow u) \in E$ must exist.

*Neighbors* The vertices of an edge are neighbors of each other. Therefore, for a node, there can be more than one neighbor depending on how many edges it belong to. The neighbors of a node *u* are defined as $N(u)=\{v|(u,v) \in E \}$. Directly connected neighbors are called first-order neighbors or level-0 neighbors. Neighbors of neighbors are termed as second-order neighbors or level-1 neighbors.

*Degree* The degree of a node in a graph is the number of its connected edges. The degree of a node *u* in a graph *G* is denoted $deg(u)=|N(u)|$. Here, *N*(*u*) is the directly connected neighbors.

*Average degree* The average degree of a graph $G = (V,E)$ is a measure of how many edges are in the set *E* compared to the number of vertices in the set *V*. The average degree of a graph $G = (V, E)$ is defined by $Avgdeg = 2 {\left| {E} \right| } / {\left| {V} \right| }$.

### Function annotation using GrAPFI-GO

GrAPFIGO is a neighborhood-based GO annotation approach that works on a network of proteins linked through domain and family information. The domain is an evolutionarily conserved region of protein sequences. A long enough protein sequence can have multiple conserved regions or domains that make up the domain composition. GrAPFI-GO follows the following steps to perform functional annotation (as illustrated in [1, 2]).

Firstly, it builds a graph by using data from protein database. Each node *u* in the graph represents a protein. An edge (*u*, *v*) between two nodes/proteins *u* and *v* means that the linked proteins share some attributes like domains and functional sites. A node *u* may have a set of labels *L*(*u*) (one or more GO annotations to propagate), has a set of neighbors *N*(*u*), and for every neighbor $v\in N(u)$, it has an associated link weight $W_{u,v}$. The overall aim is to propagate labels (*i.e.* annotations) from nodes having labels to similar nodes that lack labels. Jaccard similarity index is used to compute the link weight as $W_{P1,P2}=\frac{|D1 \cap D2|}{|D1 \cup D2|}$ for two proteins P1 and P2 having sets of domains *D*1 and *D*2, respectively.

For instance, let us consider we have five annotated proteins: *P*1, *P*2, *P*3, *P*4, and *P*5. The domain compositions of these proteins are $D1=(d1,d2,d3,d4)$, $D2=(d1,d3,d5)$, $D3=(d1,d2,d10)$, $D4=(d5,d6,d1)$, and $D5=(d4,d1,d10,d40, d7, d9, d12, d52, d100)$, respectively.

Domain composition is the set of domains arranged without order of appearance in the amino acid sequence. For example, the domain information may come in ordered form like $D1=(d1,d2,d3,d4)$ or in un-ordered form like $D1=(d1,d4,d3,d2)$. Therefore, the composition is not strictly linear. Moreover, overlap between domains is ignored as long as uniquely identified domains contained in the domain composition set.

Using the Jaccard similarity index, the link weight between P1 and P2 is calculated as,

$$\begin{aligned} W_{P1,P2}=\frac{|(d1,d2,d3,d4) \cap (d1,d3,d5)|}{|(d1,d2,d3,d4) \cup (d1,d3,d5)|}=\frac{|(d1, d3)|}{|(d1,d2,d3,d4,d5)|}=\frac{2}{5} = 0.4. \end{aligned}$$

Similarly, for P1 and P5, the link weight is calculated as,

$$\begin{aligned} W_{P1,P5}= \frac{|(d1,d2,d3,d4)\cap (d4,d1,d10,d40, d7, d9, d12, d52,d100)|}{|(d1,d2,d3,d4)\cup (d4,d1,d10,d40, d7, d9, d12, d52,d100)|}= \frac{2}{11} = 0.18. \end{aligned}$$

The rationale for using Jaccard similarity can be explained using the above example. The similarity in terms of shared domains is the same for P1 and P2, and for P1 and P5, namely 2 shared domains, *d*1 and *d*3. However, in the former case, there are only 5 different domains of which 2 are shared, whereas in the later case there are 11 different domains in total. Although two domains are shared in each case, P1 is intuitively more aligned with P2 than P5. Therefore, instead of using the raw similarity score, we used the Jaccard similarity index that better reflects the proximity of two proteins in terms of domain composition.

Secondly, a label propagation approach is applied to the protein graph in order to infer functional properties of the unlabeled nodes. Given a query protein, based on the domains and family information it contains all the neighboring proteins and their annotations are retrieved from the weighted graph. After getting the neighbors, all labels of the neighbor proteins are weighted with the edge weight that these neighbors exhibit with the query protein. When retrieving neighbors, it is possible to select only those neighbors which meet a certain similarity threshold. This means that the links can be filtered based on a predefined cut-off weight. For each candidate annotation, GrAPFI provides a confidence score, named model score (MS), that is computed as:

1 $$\begin{aligned} MS(u,i)=\frac{\sum _{v \in N(u)} W_{u,v} \sum _{j \in L(v)}\delta (j, i) }{\sum _{v \in N(u)} W_{u,v}} \end{aligned}$$

where *MS*(*u*, *i*) is the weighted score of the candidate function *i* for the query protein *u*. And $\delta (j, i)$ is 1 if the function *j* of the protein v is the same as the candidate function i, otherwise, 0.

The protein similarity graph contains both annotated and unannotated proteins. The objective of the label propagation algorithm is to transfer the annotation from annotated proteins to the unannotated proteins. To illustrate the algorithm, let us take an unannotated protein *P* with a set of domains $D=(d5, d6, d101)$. According to the Jaccard similarity, *P* is connected with *P*2 and *P*4 in the domain similarity graph. Therefore, the protein *P* will have *P*2 and *P*4 as neighbors *i.e.* $N(P)=\{P2,P4\}$. As soon as *N*(*P*) is computed, the GO annotations from neighbor proteins are propagated along with the corresponding weights computed using Eq. 1. During label propagation, we do not count the unannotated proteins that appear in *N*(*p*). All of the functional annotations are ranked based on their cumulative weights. The top-ranked annotations are selected as the best functional annotations for protein *P*.

### Node similarity based on common neighbors

GrAPFI [1] propagates the labels based on the domain similarity. In the extended version in GrAPFI-GO, we also incorporate the node similarity based on common neighbors. The labels are propagated according to the aggregated score of link weight and neighbor similarity.

We explore following eight node similarity approaches based on common neighbors. These approaches uses node neighborhood-related structural information to compute the similarity of each node with other nodes in the network. A detail description of the approaches can be found in [55].

*Common neighbors (CN)* computes the similarity as,

2 $$\begin{aligned} S(p1,p2)=|N(p1) \cap N(p2)| \end{aligned}$$

for two proteins *p*1 and *p*2 in the domain similarity graph.

*The Jaccard index (JA)* computes the node similarity as,

3 $$\begin{aligned} S(p1,p2)=\frac{|N(p1) \cap N(p2)|}{|N(p1) \cup N(p2)|} \end{aligned}$$

*The preferential attachment index (PA)* computes the node similarity as,

4 $$\begin{aligned} S(p1,p2)=|N(p1)|*|N(p2)| \end{aligned}$$

*The Salton index (SA)* computes the node similarity as,

5 $$\begin{aligned} S(p1,p2)=\frac{|N(p1) \cap N(p2)|}{ \sqrt{|N(p1)| |N(p2)|} } \end{aligned}$$

*The Sorensen index (SO)* computes the node similarity as,

6 $$\begin{aligned} S(p1,p2)=\frac{2|N(p1) \cap N(p2)|}{ |N(p1)|+ |N(p2)| } \end{aligned}$$

*The hub promoted index (HPI)* computes the node similarity as,

7 $$\begin{aligned} S(p1,p2)=\frac{|N(p1) \cap N(p2)|}{ min (|N(p1)|, |N(p2)|) } \end{aligned}$$

*The hub depressed index (HDI)* computes the node similarity as,

8 $$\begin{aligned} S(p1,p2)=\frac{|N(p1) \cap N(p2)|}{ max (|N(p1)|, |N(p2)|) } \end{aligned}$$

*The local Leicht–Holme–Newman index (LLHN) * computes the node similarity as,

9 $$\begin{aligned} S(p1,p2)=\frac{|N(p1) \cap N(p2)|}{ |N(p1)| |N(p2)| } \end{aligned}$$

As stated in [55], the aforementioned node similarity methods are simple in definition and effective in performance. All of them compute the similarity based on first-order common neighbors. Other methods that look for 2nd-order neighbors are computationally expensive. Therefore, we left those approaches for future exploration. During the label propagation, depending on the similarity measure, we take *S*(*p*1, *p*2) in Eqs. 2 to 9, and add it to the non-normalized model score. In essence, the combined score (CS) follows the equation given below for two proteins *u* and *v* (Eq. 10):

10 $$\begin{aligned} CS(u,i)=\sum _{v \in N(u)} S(u,v) \sum _{j \in L(v)}\delta (j, i) + \sum _{v \in N(u)} W_{u,v} \sum _{j \in L(v)}\delta (j, i) \end{aligned}$$

For a set of propagated labels *J* for the query protein *u*, the normalized combined score is found as,

$$\begin{aligned} \frac{CS(u,i)}{max(CS(u,i) \forall i \in J)} \end{aligned}$$

Based on this score, the top-scored annotations are recorded for performance evaluation.

### Pruning prediction set using semantic similarity score

We observed that the state-of-the-art tools in the field of GO annotation [56, 57] yield a large number of predictions. Due to the large number of predicted annotations for each protein, precision of the model declines while recall increases. However, results from these approaches raise a big concern on false positives in the predictions. Therefore, we need a method that increases the precision of the model, and hence decreases false positives in the predicted set.

To reduce the number of false positive annotations, we adopted a naive pruning technique by identifying and eliminating the outlier annotations using semantic similarity. Measuring semantic similarity between GO terms has always been an essential step in functional bioinformatics research. In a set of predicted GO annotations for a protein, pairwise semantic similarity between GO terms can show how closely these terms are related to each other and not just to the protein. We used an open-source tool called GOGO [51] for calculating the functional similarity score between GO terms and thus used it to compute the membership score of each predicted GO terms.

GOGO is a relatively fast method which does not need to calculate the information content (IC) from a large gene annotation corpus. It rather considers the number of children nodes in the GO DAG when calculating the semantic contribution of an ancestor node toward its descendent nodes. GOGO is based on GO DAG topology instead of IC which means that it is comparatively stable.

Given $DAG_g=(g,T_g, E_g)$, the Directed Acyclic GO Graph of a term *g*, its ancestors set $T_g$, and $E_g$ the set of edges between terms in $T_g$, the weight of semantic contribution is calculated as

11 $$\begin{aligned} w_e(t)=1/(c+nc(t))+d, \end{aligned}$$

where c and d are constants determined by empirical observations, and *nc*(*t*) is the total number of children of term $t\in T_g$. The semantic contribution of each term in $DAG_g=(g,T_g, E_g)$ is defined as,

12 $$\begin{aligned} S_g(t) = \left\{ \begin{array}{cc} 1 &{} \quad if \, t=g \\ max\{w_e(t)*S_g(t')|t'\in children(t)\} &{} \quad if \, t\ne g \\ \end{array} \right. \end{aligned}$$

Therefore, the aggregated semantic value for the term *g* is computed as

13 $$\begin{aligned} SV(g)=\sum _{t\in T_g}{S_g(t)}. \end{aligned}$$

In the case of two terms *g*1 and *g*, having $DAG_{g1}=(g1, T_{g1}, E_{g1})$ and $DAG_{g2}=(g2, T_{g2}, E_{g2})$, the semantic similarity between *g*1 and *g* is as follows:

14 $$\begin{aligned} SS(g1,g2)=\frac{\sum _{t \in T_{g1} \cap T_{g2}} (S_{g1}(t)+S_{g2}(t))}{SV(g1)+SV(g2)}. \end{aligned}$$

Finally, the functional similarity between a set of GO terms, $A= \{g1, g2, g3,...,gm\}$ and a query GO term $g\notin A$ and is as follows:

15 $$\begin{aligned} SS(g,A)= max_{1 \le i \le m } (SS(g,g_i\in A)). \end{aligned}$$

We use Eq. 15 to calculate the semantic similarity between each pair of GO terms in the predicted set of annotations *A*. Then, we can compute the membership of each annotation in set *A* as follows:

16 $$\begin{aligned} SS(g_i,A)= max_{1 \le j \le m } (SS(g_i\in A,g_j\in A\setminus \{g_i\})), \end{aligned}$$

Instead of maximum, membership score can also be calculated as the average and Root Mean Square (RMS) score of each annotation in the set. For this study, we used RMS score as it gave the best results. We name this measure of membership as semantic similarity (SS) score.

### Aggregation of scores

In all state-of-the-art GO annotation models, used for experiments in this study, there is a prediction score associated with each predicted annotation for every protein. We refer to this as model score (MS).

For a protein, *u* with a set of predicted annotations *A*, each annotation $g\in A$ has two scores associated to it: (1) first, the Model Score (MS) , defined as *MS*(*u*, *g*), which shows the credibility with which the annotation was predicted by a particular annotation tool and (2) second, the Semantic Similarity (SS) score, defined as $SS_u(g,A)$, which shows the semantic similarity of each member annotation *g* to the predicted set *A*. Now, we need to combine these scores to find a combined prediction (CP) score, defined as $CP_u(g,A)$, for each annotation $g \in A$ of protein *u*. Joining the scores into a single score provides an overall assessment. A score should be able to distinguish between annotations that score average in both MS score and SS score, from those that score high in one scoring scheme and low in the other scheme. Therefore, instead of averaging the scores, we follow the following scheme:

17 $$\begin{aligned} CP_u(g,A) = \sqrt{ \frac{\left( \frac{MS(u,g)}{max\_MS}\right) ^2 + \left( \frac{SS_u(g,A)}{max\_SS}\right) ^2 }{2} } \end{aligned}$$

Here, $max\_MS$ and $max\_SS$ denotes the maximal model score and semantic similarity score, respectively. The range for the two scores is therefore bounded from 0 to 1. Since this is a technique to prune an already predicted set, we take the square root in the equation to increase the overall value of the combined scores, so as to increase the threshold values. Once we have the combined score, we can define a certain score as cutoff to filter the predicted set. Annotations with scores above the cutoff form a new set of predicted annotations.

### Hierarchical post-processing

The final step of the process is hierarchical post-processing of predictions in the new predicted set. In the Gene Ontology DAGs, the GO terms hold various parent–child relations putting biologically closer GO terms hierarchically nearer in the graph. We implemented a methodology to include more complete predictions by including the ancestors of all the GO terms in the new set of predictions. The ancestors of a GO term in the DAG it belongs to, have a very high semantic similarity with the term. Therefore, we first topologically sorted the DAG for each GO aspect and determined all possible paths from each GO term to the root of the corresponding aspect. Finally, we follow these paths from terms to the root, one by one and add corresponding ancestors to the set of predictions to obtain the final prediction set.

## Data Availability

The data and materials can be accessed here: https://github.com/Bishnukuet/GrAPFI-GO.git.

## Abbreviations

GrAPFI: Graph-based automatic protein function inference
GrAPFI-GO: Graph-based automatic protein function inference for GO annotation
EC: Enzyme Commission
GO: Gene ontology
HMM: Hidden Markov model
BLAST: Basic local alignment search tool
PSI-BLAST: Position specific iterative basic local alignment search tool
SAAS: Statistical automatic annotation system
PPI: Protein protein interaction
PSSM: Position specific scoring matrix
FDR: Functionally discriminating residues

## Footnotes

1 https://www.biofunctionprediction.org/cafa/.
